# Zosteriform cutaneous metastases: an unusual presentation of metastatic lung carcinoma

**DOI:** 10.1002/rcr2.515

**Published:** 2019-12-18

**Authors:** Kirsty J. L. Wark, Melani Mahendran, Artiene Tatian, Amandeep Singh, Jane Woods, Ananthakrishnapuram Aravindan

**Affiliations:** ^1^ Department of Dermatology Liverpool Hospital Liverpool NSW Australia; ^2^ Ingham Institute of Applied Medical Sciences Liverpool NSW Australia; ^3^ School of Medicine University of New South Wales Liverpool NSW Australia; ^4^ Department of Medicine Liverpool Hospital Liverpool NSW Australia; ^5^ School of Medicine Western Sydney University Liverpool NSW Australia; ^6^ Department of Anatomical Pathology Liverpool Hospital Liverpool NSW Australia; ^7^ Department of Renal Medicine Liverpool Hospital Liverpool NSW Australia

**Keywords:** Lung adenocarcinoma, metastatic adenocarcinoma, renal disease, vascular catheter and zosteriform cutaneous metastases

## Abstract

Zosteriform cutaneous metastases are an unusual and rare morphological variant. We discuss the case of a 78‐year‐old gentleman with a background of end‐stage renal disease with metastatic adenocarcinoma of the lung which was diagnosed due to the development of zosteriform cutaneous metastases around his vascular catheter (vascath) site. The vascath may have acted as a traumatic nidus for lymphatic spread.

## Graphical abstract

Zosteriform cutaneous metastases are a rare morphological variant. We present a case of metastatic adenocarcinoma of the lung in a 78‐year‐old presenting as zosteriform cutaneous metastases around a vascular catheter site.

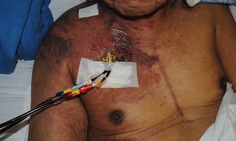

## Introduction

Zosteriform cutaneous metastases are a rare morphological variant, which is infrequently described in the literature. In this paper, we report a case of a 78‐year‐old gentleman with zosteriform cutaneous metastases of lung adenocarcinoma, associated with a right internal jugular vascular catheter (vascath) site in a patient with end‐stage renal disease (ESRD).

## Case Report

A 78‐year‐old gentleman of South‐East Asian heritage was admitted with cervical lymphadenopathy and a three‐week history of a new rash for investigation. He had a background of ESRD and was on haemodialysis via a right internal jugular vascath which had been inserted four to five months prior. His other past medical history included ischaemic heart disease with previous coronary artery bypass grafting, prior left frontal cardiovascular cerebral accident, atrial fibrillation, hypercholesterolaemia, and hypertension. He was HIV negative, was not immunosuppressed, and was not a transplant recipient. He was a reformed smoker, with a 25‐year pack history.

**Figure 1:**
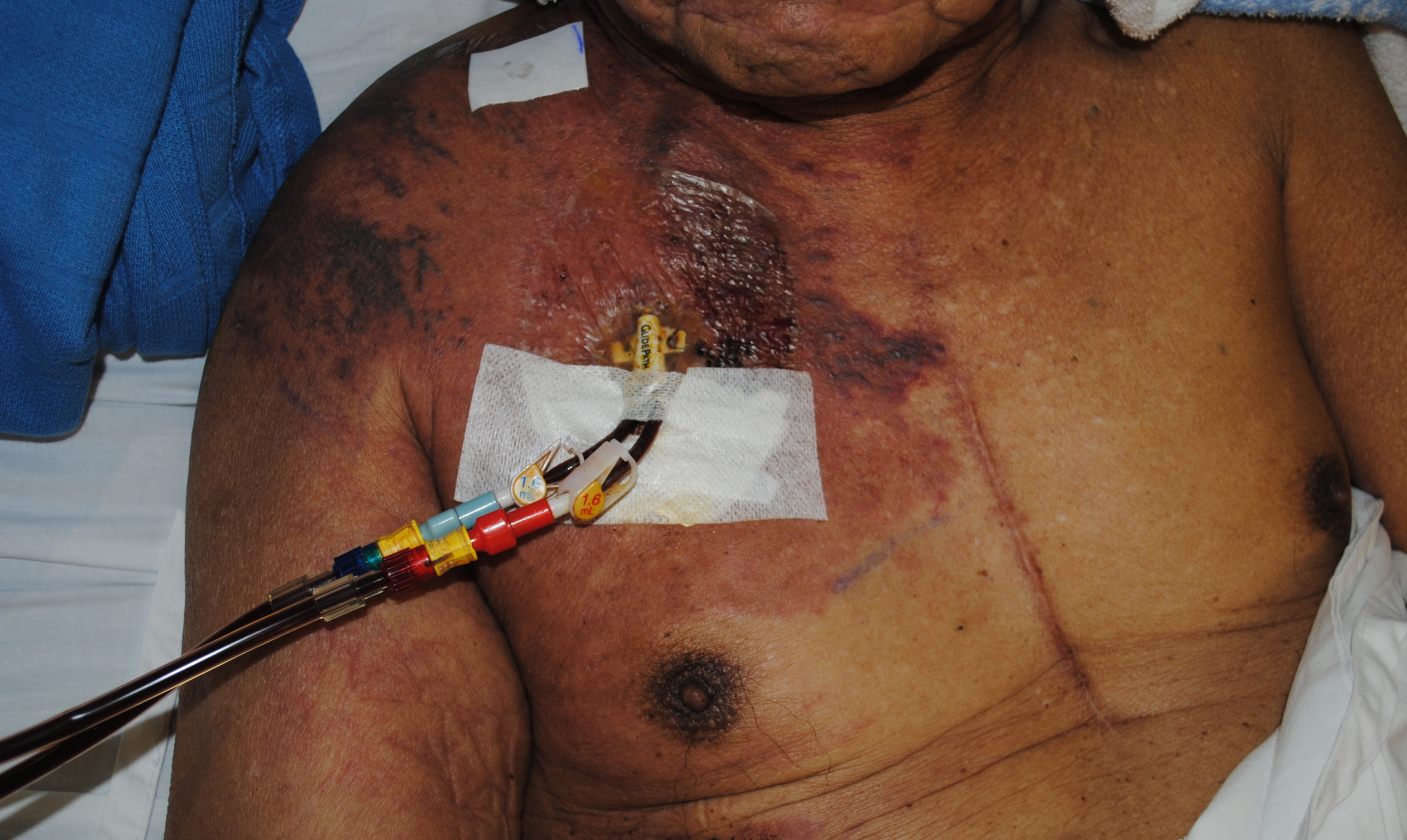
Zosteriform erythematous to violaceous plaques over the right chest, shoulder, abdomen, and neck with overlying microvesicles and dilated capillaries.

The rash had started around the vascath site and had spread rapidly to his midline, right shoulder, and neck, as well as being associated with a similar isolated patch on his right abdomen. It was non‐pruritic and non‐painful. On examination, he had zosteriform erythematous to violaceous plaques with overlying microvesicles and dilated capillaries (Figure 1). There was a sharp mid‐chest demarcation along an old sternotomy scar. He also had bilateral cervical and right‐sided axillary lymphadenopathy. Varicella zoster and herpes simplex viral swabs were negative.

**Figure 2:**
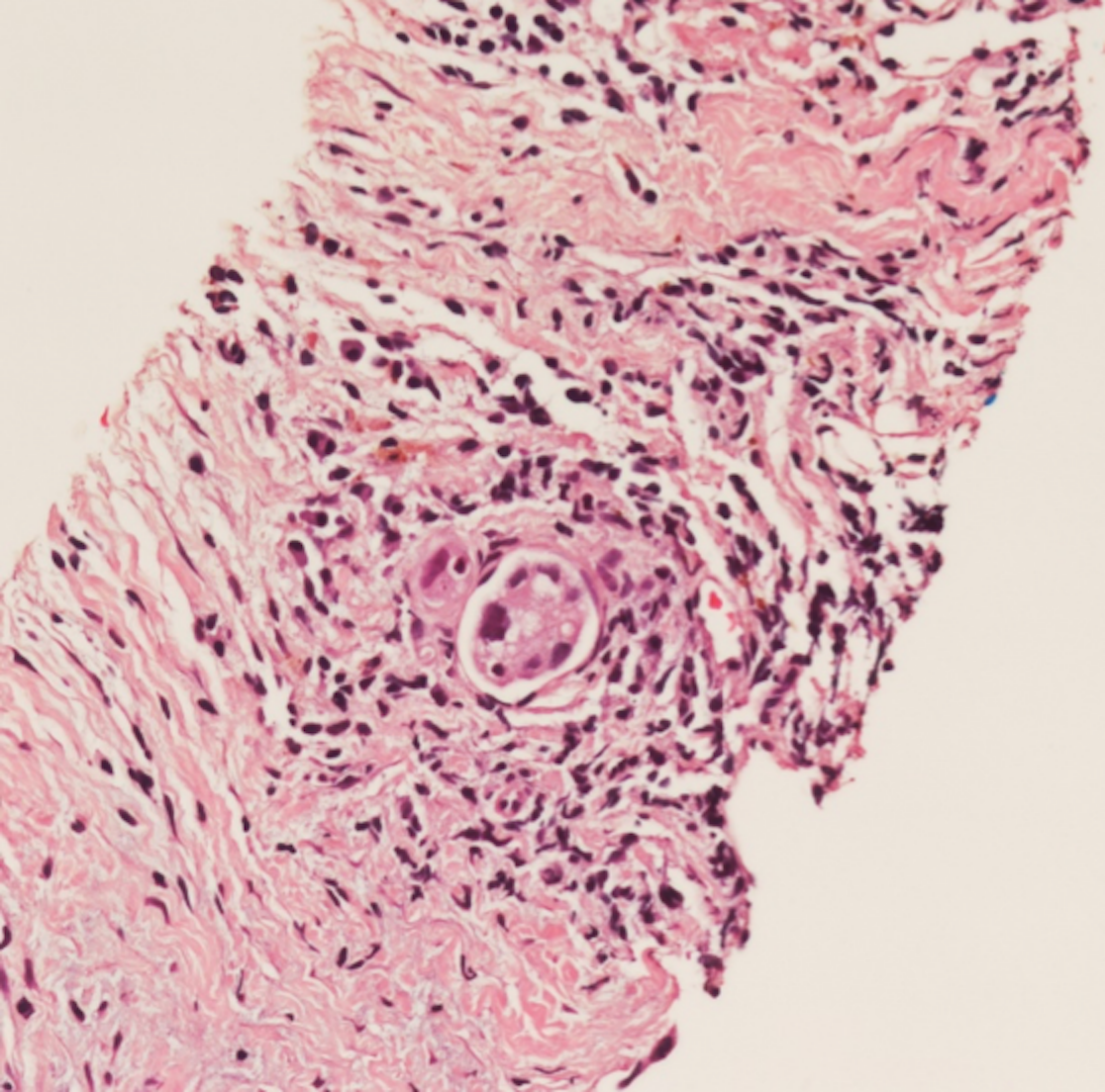
Dermal lymphatics with carcinoma. There is surrounding chronic lymphoplasmacytic infiltrate (haematoxylin and eosin (H&E): 200×).

Abdominal skin and right supraclavicular lymph node biopsies were performed, and both showed metastatic non‐small cell carcinoma, with positive cytokeratin (MNF116) and Thyroid transcription factor 1 (TTF1) immunostaining favouring an adenocarcinoma of lung origin. The skin biopsy showed a lymphatic pattern of distribution of the adenocarcinoma in keeping with its metastatic nature (Figure 2). Endothelial growth factor receptor (EGFR), anaplastic lymphoma kinase (ALK), and repressor of silencing 1 (ROS1) were negative, and programmed cell death ligand (PD‐LI) expression score was <1% (none). A computer tomography scan of his chest was performed, which revealed a well‐circumscribed soft tissue opacity in the right upper lobe measuring 1.7 × 1.4 × 2.1 cm, and a smaller anterior nodule measuring 1.2 × 1 × 1 cm. A diagnosis of metastatic lung adenocarcinoma was made. The clinical decision was made not to proceed with positron emission tomography (PET) imaging, as this would not change management. He was explained about the advanced nature of his malignancy. After discussions with various specialities, the patient and family opted for conservative management of the malignancy. He developed malignant pleural effusion few weeks later and passed away.

### Discussion

Cutaneous metastases are estimated to occur in approximately 2.1% of patients with lung cancer, and in about half of these cases are the first sign of extranodal disease 1. Zosteriform cutaneous metastases are a rare presentation, and are defined by their distribution along dermatomes rather than their morphology 2. One large retrospective study identified that in metastatic lung cancer with skin metastases, the majority were distant lesions from the thorax 1.

Several mechanisms have been proposed to explain zosteriform metastases, including a Koebner or Koebner‐like phenomenon in skin previously affected by the varicella zoster virus (VZV), or post‐herpetic neural and immunological modulation which predisposes the previously affected skin to metastases 2, 3.

A previous case report describes an incisional cutaneous metastasis in a patient with large cell adenocarcinoma of the lung nine months after a posterolateral thoracotomy and bilobectomy 4. Whilst in that case the incisional site was from surgical management of the malignancy, a separate case of colonic adenocarcinoma occurring as a cutaneous metastasis in an old cholecystectomy scar has been described 5.

In our case, the vascath site may have functioned as a traumatic nidus for cutaneous metastasis, likely through lymphatic spread based on the histology findings and presence of lymphadenopathy. This is supported by the history of the rash, which started initially on the skin around the vascath. However, what is not known is whether this patient previously had VZV infection in the affected areas. Therefore, the possible role of VZV infection in predisposing the affected sites cannot be established with any certainty. Alternatively, haematogenous spread is also possible given the vascath is in direct communication with the inferior internal jugular vein but appears to be a less likely explanation given the associated regional lymph node spread.

### Disclosure Statement

Appropriate written informed consent was obtained for publication of this case report and accompanying images.

K. J. L. Wark is a relative of Professor Peter Wark who is on the editorial board.
